# Dilemmas of evaluation: health research capacity initiatives

**DOI:** 10.2471/BLT.14.141259

**Published:** 2014-10-10

**Authors:** Donald C Cole, Garry Aslanyan, Alison Dunn, Alan Boyd, Imelda Bates

**Affiliations:** a Dalla Lana School of Public Health, University of Toronto, Health Sciences Building (room 676), 155 College Street, Toronto, Ontario, M5T 3M7, Canada.; b World Health Organization, Geneva, Switzerland.; c Write Space, Brighton, England.; d Manchester Business School, University of Manchester, Manchester, England.; e Liverpool School of Tropical Medicine, Liverpool, England.

Strengthening health research capacity in low- and middle-income countries (LMIC) is a recognized way to advance health and development. Numerous approaches to strengthening capacity exist at different levels, including training for individuals, improving research systems within institutions, and international collaborations among national health research agencies.^1^ Systematic evidence on the effectiveness of different approaches remains limited, as their complexity and diversity make monitoring and evaluation difficult. Assessing returns on investments on research capacity strengthening has been challenging, since many funders have given low priority to rigorous monitoring and evaluation.

In 2008 funding agencies came together as the ESSENCE on Health Research initiative. This group realized that there were too many different forms of monitoring and evaluation for health research capacity strengthening. It wanted to reduce the administrative burden on funding recipients, to enable learning and improvement among stakeholders and to demonstrate the impact of research capacity strengthening projects. In 2011, ESSENCE published a Planning, Monitoring and Evaluation Framework as a guide for its member funding agencies and grantees. The framework encouraged the sharing of lessons about evaluations of these projects.

We were interested in understanding different approaches to health research capacity strengthening and analysed evaluation reports of health interventions funded by ESSENCE members and other agencies. This perspective is based on analyses of the funders’ reports.^2^^–^^4^

## Addressing tensions

We found that a critical tension facing funders and policy-makers is the degree to which recipients of funding should be involved in the evaluation of their own projects. Some funders perceive that an externally-led evaluation better demonstrates accountability and value for money. Funders noted that some recipients had limited involvement in – or capacity for – evaluations, lacking critical skills such as the ability to set testable goals and measurable targets.

However, when recipients participate in evaluations from conception to completion, they have a greater sense of ownership over the project and are more likely to learn from their work. They are also able to make ongoing improvements to the project in the context of developmental or empowerment-type evaluations and can subsequently implement recommendations leading to long-term change. Compared to external evaluators, recipients usually have greater in-depth knowledge about the project, the context and other stakeholders. This knowledge is vital for solving problems. Recipients who are involved in the evaluation can help the rest of the team detect and correct problems early on, communicate decisions and act on results. Engaging other stakeholders – such as service users, community members, health practitioners and policy-makers – is helpful for setting realistic goals, meeting local priorities and addressing resource issues. This requires extensive participation and hence more resources.

We recommend that funders and recipients jointly agree on the purpose and process of the evaluation, and help stakeholders to fulfil their roles in evaluations of research capacity strengthening. Among their portfolios of projects and programmes, funders can up-front commission evaluations, participate in them and subsequently use evaluation results. Funding recipients can design monitoring and evaluation processes and generate data. Evaluation teams including external evaluators and recipients are ideally composed of people with technical, organisational and interpersonal skills. Their understanding of the complexity of research capacity strengthening can enrich the evaluations they undertake.^2^

## Theory-based frameworks

Capacity strengthening is a complex process with a long intervention pathway. Applying a theory of change can help to define the pathway and inform the evaluation.^5^ It can help to identify impact trajectories, strengthen evaluation rigour, foster assessment of generalizability to other contexts, and guide influence on policy and practice.

Theory-driven evaluative thinking can lead to more useful evaluations, as stakeholders try to identify underlying assumptions and rationale for their actions. However, most evaluations do not have the time and resources to incorporate theory-informed indicators of impact and sustainability, or to collect data against these indicators. This means missed opportunities to enhance knowledge and learning among funders and funding recipients about how to improve planning, monitoring and evaluation of research capacity initiatives.

A key recommendation from our research is for projects to develop comprehensive, prospective systems for research capacity evaluation. Funders and policy-makers could develop supporting guidance, tools or training for such evaluations and allocate adequate funding to evaluations.^3^

## Indicators

A systematic analysis of assumptions, preconditions and measurement options associated with an evaluation can make monitoring processes easier. Although a few common measurable, reliable indicators may be feasible, most projects will need some indicators tailored to the context and the project objectives. Baseline measurement of selected indicators would enable a prospective, rather than the much less rigorous, retrospective approach.

It is important to maintain flexibility and revisit indicators as the research capacity project proceeds.^1^ Evaluations should distinguish between the provincial-national research environment, the international-global environment and research networks. This would help facilitate greater clarity of relationships in pathways of change and consistency in cross-case comparisons.^4^

## Evaluation as a learning tool

There are different elements to evaluation as a learning tool. Broader lessons can be shared among the health research capacity community (Box 1). Skills learnt by individuals during evaluation of projects can enhance their knowledge and ability to plan, fund, or implement better research capacity strengthening programmes.

Box 1 **Improving the monitoring and evaluation of health research capacity strengthening**

**An example from the International Centre for Diarrhoeal Disease Research in Bangladesh (ICDDR,B)^6^**

ICDDR,B sought to strengthen its health research capacity and designed a new monitoring and evaluation framework. It was theory-based and built on six principles of health research capacity strengthening. Experts from a dedicated monitoring and evaluation unit were engaged in the project.

Involvement of funders increased inter-institutional collaboration and produced efficiencies, but initially alienated researchers who saw few benefits from monitoring and evaluation. The experts responded to the researchers by modifying the comprehensive system of indicators underpinned by good quality data which met multiple stakeholder needs.

The monitoring and evaluation system was flexible, responsive to changes in the organization’s focus, and inclusive of evaluation data processing capabilities. Findings from monitoring and evaluation have informed ICDDR,B’s ongoing research and evaluation strategy.

Donor governments often encourage research capacity strengthening initiatives to garner additional resources, so that the initiatives become self-sustaining. Developmental or empowerment evaluations can contribute to sustainability through the learning achieved. Evaluators are well positioned to facilitate discussions among stakeholders and promote empowerment by enhancing stakeholders’ skills to address tensions, negotiate approaches to problems and come to decisions. Funders could also support the development of a community of practice to share lessons and experiences of research capacity strengthening and to consider joint ways forward.^5^

## Conclusion

Funders and policy-makers aiming to harmonize evaluation approaches for health research capacity strengthening initiatives must successfully manage underlying tensions to move forward. These include the degree of stakeholder participation, the right balance of quantitative and qualitative data, the promotion of learning while gathering information and an emphasis on long-term, as well as short-term, gains. A deeper analysis of health research capacity strengthening projects, using consistent and multiple methods would enable learning to be shared and transferred. It would also relieve funding recipients of the burden of multiple reporting, consistent with aid effectiveness principles, and potentially enable funders to better demonstrate impact and value for money.
